# Aerobic exercise training engages the canonical wnt pathway to improve pulmonary function and inflammation in COPD

**DOI:** 10.1186/s12890-024-03048-z

**Published:** 2024-05-14

**Authors:** Peijun Li, Xiaoyu Han, Jian Li, Yingqi Wang, Yuanyuan Cao, Weibing Wu, Xiaodan Liu

**Affiliations:** 1 School of Rehabilitation Science, Shanghai University of Traditional Chinese Medicine, Shanghai, 201203 P.R. China; 2 Department of Sports Rehabilitation, Shanghai University of Sport, Shanghai, 200438 P.R. China; 3 Faculty of Traditional Chinese Medicine, Naval Medical University (Second Military Medical University), Shanghai, 200433 P.R. China; 4 Institute of Rehabilitation Medicine, Shanghai Academy of Traditional Chinese Medicine, Shanghai, 201203 P.R. China; 5 Engineering Research Center of Traditional Chinese Medicine Intelligent Rehabilitation, Ministry of Education, Shanghai, 201203 P.R. China

**Keywords:** Chronic obstructive pulmonary disease, Treadmill exercise, Pulmonary function, Inflammation, Cigarette smoke, Wnt/β-catenin

## Abstract

**Background:**

We studied whether the exercise improves cigarette smoke (CS) induced chronic obstructive pulmonary disease (COPD) in mice through inhibition of inflammation mediated by Wnt/β-catenin-peroxisome proliferator-activated receptor (PPAR) γ signaling.

**Methods:**

Firstly, we observed the effect of exercise on pulmonary inflammation, lung function, and Wnt/β-catenin-PPARγ. A total of 30 male C57BL/6J mice were divided into the control group (CG), smoke group (SG), low-intensity exercise group (LEG), moderate-intensity exercise group (MEG), and high-intensity exercise group (HEG). All the groups, except for CG, underwent whole-body progressive exposure to CS for 25 weeks. Then, we assessed the maximal exercise capacity of mice from the LEG, MEG, and HEG, and performed an 8-week treadmill exercise intervention. Then, we used LiCl (Wnt/β-catenin agonist) and XAV939 (Wnt/β-catenin antagonist) to investigate whether Wnt/β-catenin-PPARγ pathway played a role in the improvement of COPD via exercise. Male C57BL/6J mice were randomly divided into six groups (*n* = 6 per group): CG, SG, LiCl group, LiCl and exercise group, XAV939 group, and XAV939 and exercise group. Mice except those in the CG were exposed to CS, and those in the exercise groups were subjected to moderate-intensity exercise training. All the mice were subjected to lung function test, lung histological assessment, and analysis of inflammatory markers in the bronchoalveolar lavage fluid, as well as detection of Wnt1, β-catenin and PPARγ proteins in the lung tissue.

**Results:**

Exercise of various intensities alleviated lung structural changes, pulmonary function and inflammation in COPD, with moderate-intensity exercise exhibiting significant and comprehensive effects on the alleviation of pulmonary inflammation and improvement of lung function. Low-, moderate-, and high-intensity exercise decreased β-catenin levels and increased those of PPARγ significantly, and only moderate-intensity exercise reduced the level of Wnt1 protein. Moderate-intensity exercise relieved the inflammation aggravated by Wnt agonist. Wnt antagonist combined with moderate-intensity exercise increased the levels of PPARγ, which may explain the highest improvement of pulmonary function observed in this group.

**Conclusions:**

Exercise effectively decreases COPD pulmonary inflammation and improves pulmonary function. The beneficial role of exercise may be exerted through Wnt/β-catenin-PPARγ pathway.

**Supplementary Information:**

The online version contains supplementary material available at 10.1186/s12890-024-03048-z.

## Introduction

Chronic obstructive pulmonary disease (COPD) is ranked as the third cause of the mortality all over the world [1]. It is a common disease involving maladjusted pulmonary inflammation, characterized by bronchiolitis and emphysema [2]. Cigarette smoke (CS) is a significant risk factor for COPD development. It can give rise to elevated pulmonary inflammation, including increased tumor necrosis factor (TNF-α), interleukin (IL)-6, IL-8, and IL-1β [3 – 5], and release of proteolytic enzymes, including neutrophil elastase, matrix metalloproteinases, and myeloperoxidase. These factors lead to changes in the pulmonary structure and function, including alveolar fracture, alveolar space enlargement and airway remodeling [6, 7]. Pulmonary inflammation shows an association with disease progression and increased mortality in COPD patients [8, 9].

Canonical Wnt/β-catenin signaling plays a role in pulmonary development (neoangiogenesis and lung branching morphogenesis), growth, tissue regeneration and inflammatory responses [10]. Extracellular Wnt ligands bind to transmembrane receptors. This condition leads to the formation of a “destruction complex” comprising adenomatous polyposis coli protein, axis inhibition protein, casein kinase 1 and glycogen synthase kinase 3 protein (GSK3), which inhibits the process of β-catenin degradation. β-catenin translocates to the nucleus and activates downstream target genes by binding with T cell factor (TCF)/lymphocyte enhancer-binding factor (LEF) 1 [10 – 12]. The level of nuclear-β-catenin expression in airway epithelial cells increases in COPD patients, which is negatively correlated with disease severity [10]. Inhibition of Wnt/β-catenin causes increased ciliated cell numbers, epithelial polarity, and barrier function, and a decreased epithelial-to-mesenchymal transition [10]. The TCF/LEF domain in β-catenin can directly interact with and inhibit peroxisome proliferator-activated receptor (PPAR) γ [13], which results in the mediation of pulmonary senescence [14]. Moreover, PPARγ is a ligand-dependent inflammatory factor. PPARγ is reduced in COPD sputum compared with that from healthy controls and is positively correlated with forced expiratory volume in the first second (FEV1)/forced vital capacity (FVC), which is a diagnostic parameter according to GOLD guidelines for COPD [2, 15]. In smoke-induced mouse models, rosiglitazone and pioglitazone alleviated neutrophil infiltration in the airway [16], and IL-2 and TNF-α were reduced in the lungs and bronchoalveolar lavage fluid (BALF) [17]. In conclusion, the Wnt/β-catenin-PPARγ pathway may represent a viable therapeutic target for mitigating inflammation associated with COPD.

As critical mean of pulmonary rehabilitation, aerobic exercise is a vital nonpharmacological method that can considerably improve lung function, fatigue, dyspnea, and health-related quality of life in individuals with COPD [18]. In addition, this form of exercise can ameliorate the inflammation in the plasma and BALF in patients with COPD, and can also alleviate emphysema-like changes in the alveoli [19 – 21]. However, the mechanism underlying the exercise-inhibited inflammation in COPD remains unclear. Exercise modulates the levels of Wnt/β-catenin, PPARγ, thereby influencing inflammation levels [22 – 25]. The role of Wnt/β-catenin-PPARγ in ameliorating pulmonary inflammation and function via exercise is uncertain. We constructed a COPD mouse model through progressive whole-body CS exposure to explore this matter. We performed an 8-week exercise intervention to detect the appropriate exercise intensity for regulating Wnt/β-catenin. Then, we used the Wnt antagonist XAV939, which maintains the integrity of the destruction complex, and agonist LiCl (which inhibits GSK3β) to elucidate the role of Wnt/β-catenin in COPD pulmonary function and lung inflammation improvement with exercise. In our study, moderate-intensity exercise showed a substantial effect on reducing pulmonary inflammation and thus can be considered an appropriate exercise intensity in clinical practice. Moreover, we provided preliminary evidence of the role of Wnt/β-catenin in alleviating COPD through exercise, which should be confirmed in further studies.

## Materials and methods

### Animals and experimental design

First, male C57BL/6J mice (6–8 weeks) were randomly divided into five groups (*n* = 6 per group): a control group (CG), a smoking group (SG), a low-intensity exercise group (LEG), a moderate-intensity exercise group (MEG), and a high-intensity exercise group (HEG). Mice in the SG and exercise intervention groups underwent progressive whole-body CS exposure for 25 weeks at 6 days/week. An 8-week aerobic exercise intervention was implemented in the exercise groups for 6 days/week for 60 min each session. The appropriate intensity was determined, and another experiment that used the Wnt antagonist XAV939 and the Wnt agonist LiCl was conducted to determine the role of Wnt/β-catenin-PPARγ signaling in the effects of exercise. Male C57BL/6J mice were randomly divided into six groups (*n* = 6 per group): CG, SG, LiCl group (LiG), LiCl and exercise group (LiEG), XAV939 group (XG), and XAV939 and exercise group (XEG). All mice except those in the CG were exposed to CS. Meanwhile, mice in the exercise groups underwent moderate-intensity exercise training under the abovementioned protocol. The doses of the agonist (LiCl, 200 mg/kg body weight) and antagonist (XAV939, 2.5 mg/kg body weight) were referred to from previous studies [26, 27]. The drugs were intraperitoneally injected into the mice 30 min before exercise once per day at 6 days/week for 8 weeks. Mice in the CG and the SG did not receive any intraperitoneal injections.

All animals were housed in a specific pathogen-free barrier facility, and were subjected to a 12-hour light/dark cycle. Mice were given free access to water and food. The environmental temperature was 21 ± 2℃, and the humidity was 60 ± 10%. The Animal Care and Use Committee of SHUTCM approved the study with an approval number (PZSHUTCM210312012). Animal studies have been reported in compliance with the guide for the care and use of laboratory animals [28] and international principles for research involving animals (ARRIVE 2.0) [29].

### CS exposure protocol

All the mice were placed in a passive smoking poisoning system (PAB-S200, Beijing Bestlab High-Tech Co., Ltd. Beijing, China). The poisoning chamber was 80 × 60 × 58 cm^3^ and was embedded with an O_2_, CO, and CO_2_ gas concentration sensor to monitor gas concentrations. The presence of two outlets ensured a normal oxygen concentration and pressure. The two inlets on the side were connected to lit cigarettes, and the CS was suctioned and conducted inside the box.

Referring to our previous study [30], we established a progressive whole-body smoke exposure protocol. In the first week, we used 10 cigarettes (Huangguoshu cigarettes, 0.9 mg nicotine, 10 mg tar, and 12 mg carbon monoxide per cigarette) for every exposure once per day. In week 2–7 week, 10 cigarettes per exposure were used twice per day. In weeks 8–13, 15 cigarettes per exposure were used twice per day. In weeks 14–25, 20 cigarettes per exposure were used twice per day. At least 4 h was allowed between each cigarette exposure.

### Treadmill aerobic training and testing

The mice were adapted to the treadmill for 6 days (5 m/min), with 10 min on the first day, followed by an additional 10 min every day after. Afterward, a maximal exercise capacity test with a 5-min warm-up (5 m/min) was implemented, followed by an increase in speed (2 m/min every 3 min) until animal exhaustion (the mice were unable to run after several mechanical stimuli) [20]. The test was repeated for three times: before the exercise test, in the week 4 and in the week 8. The maximal exercise speed of every mouse refers to the maximal exercise capacity. Each group of mice underwent aerobic exercise with 35%, 55%, and 85% maximal exercise speed, for 8 weeks, 6 days/week, once a day and for 60 min/exercise.

### Respiratory function measurements

First, 1.25% avidin tribromoethanol (0.2 mL/10 g body weight) was intraperitoneally injected into the mice for anaesthesia. A cannula was inserted after tracheotomy, and the mice were connected to a pulmonary function test system (Buxco PFT, DSI, USA) to detect the lung volume and ventilation. The respiratory flow, pressure, and volume wave were shown on a computer display. Lung volume parameters including FVC, FEV in 50 ms (FEV50), and FEV in 100 ms (FEV100), and lung ventilation parameters including minute ventilation (MV), dynamic lung compliance (Cydn), and peak expiratory flow (PEF), were measured and used in the assessment of respiratory function.

### Histological staining and morphological analysis

Lung samples were dissected and fixed for 24 h in 4% paraformaldehyde at room temperature, embedded in paraffin, and sliced into 4–6 μm sections. Staining of the sections using hematoxylin and eosin was performed as previously reported [30], and the cross-sectional area (CSA) of the alveolus was calculated. Light microscopy (OLYMPUS, Tokyo, Japan) was performed, and five nonoverlapping fields were examined for each slide for statistical analysis (Image J software, Maryland, USA). Masson trichrome staining was employed to assess the collagen deposition in the airways [31].

### Inflammatory cytokine measurement in Bronchoalveolar lavage fluid

Immediately after the respiratory function measurement, the mice were sacrificed via exsanguination, and BALF was collected after the right lung was washed with 0.5 mL of sterile saline three times [32]. The samples were centrifuged at 1200 rpm for 15 min at 4℃, and the supernatant was collected and stored at − 80 ℃. Inflammatory cytokines, including IL-1β (mouse IL-1β, EM30300S), IL-6 (mouse IL-6, EM30325S), IL-8 (mouse IL-8, EM30328S), and TNF-α (mouse TNF-α, EM30536S), were measured using enzyme-linked immunosorbent assay kits from Biowell (Shanghai, China), following the manufacturer’s specifications as previously reported [30].

### Measurement of wnt/β-catenin–PPARγ in the lung

Total proteins were extracted from the left lung tissue via radioimmunoprecipitation assay buffer (Biowell, Shanghai, China), and the protein concentration was determined using a BCA protein assay kit (Biowell, Shanghai, China). Equal amounts of proteins (25 µg Wnt1, 100 µg PPARγ, and 100 µg β-catenin) were resolved on sodium dodecyl sulfate-polyacrylamide gel electrophoresis gels and then transferred to polyvinylidene difluoride membranes. At room temperature, the membranes were blocked in 5% non-fat dried milk for 2 h. The following rabbit polyclonal antibodies were used to detect anti-Wnt1 (1:2000, Immunoway, YT4907), anti-β-catenin (1:1000, Immunoway, YT5866), anti-PPARγ (1:1000, Immunoway, YT3836), and anti-glyceraldehyde-3-phosphate dehydrogenase (GAPDH) (1:2000, Biowell, WB0197). The membranes were then incubated with anti-rabbit or anti-mouse IgG horseradish peroxidase-conjugated secondary antibodies (Biowell, WB0177/WB0176). The protein bands were visualized using enhanced chemiluminescence reagents (Biowell, WB0164) and analyzed via Quantity-One software. Band intensities were quantified using Image J software (Maryland, USA). The relative expression of each immunoreactive band was calculated by comparing it with GAPDH.

### Statistical analysis

Statistical analysis was performed using SPSS 25.0 (USA). All data are presented as means ± (standard error of the mean). The Shapiro–Wilk test was used to verify data normality. A one-way analysis of variance (ANOVA) was used to compare differences among groups, and independent t-tests were conducted to detect between-group differences. The Kruskal-Wallis H test was used for non-normality distributed data. Differences were considered significant at *p* < 0.05.

## Results

### Moderate-intensity exercise effectively alleviated COPD

**Fig. 1:**
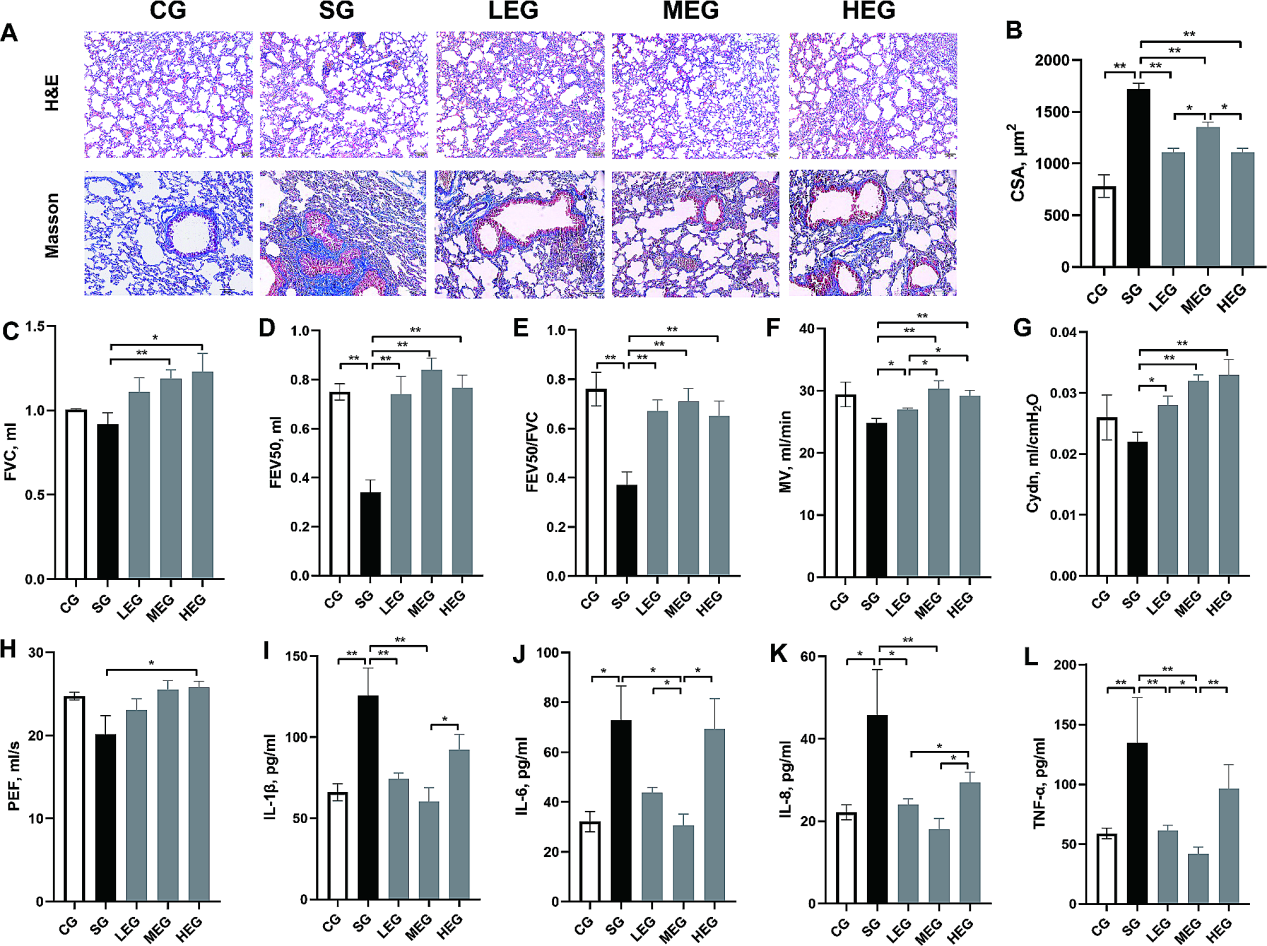
Features of COPD changes after exercise training. **A**, lung sections stained with HE and Masson (Bar = 50 μm, 100× and 200×, respectively); **B**, CSA of alveolar; **C**-**H**, parameters of lung volume and ventilation function; **I**-**L**, cytokines level of BALF. CSA, cross sectional area; FVC, forced vital capacity; FEV50, forced expiratory volume in 50 ms; MV, minute ventilation; Cydn, dynamic lung compliance; PEF, peak expiratory flow; IL, Interleukin; TNF, tumor necrosis factor; CG, control group; SG, cigarette smoke group; LEG, low-intensity exercise group; MEG, moderate-intensity exercise group; HEG, high-intensity exercise group; *p < 0.05; **p < 0.01

Exercise of various intensities caused changes in structural manifestations and functional, and inflammation levels of COPD (Fig. 1). The levels of emphysema, as indicated by the CSA, after exercise of different intensities were less severe compared with those observed in the SG (*p* < 0.05) and the collagen deposition in the airways, as revealed by Masson trichrome staining (Fig. 1A and B). The lung function results showed significant increases in FVC, FEV50, FEV50/FVC, MV, Cydn, and PEF after moderate- and (or) high-intensity exercises (compared with the SG, *p* < 0.05; Fig. 1C–H). IL-1β, IL-6, IL-8, and TNF-α showed significantly increased after CS (compared with the CG, *p* < 0.05; Fig. 1I–L), whereas IL-1β, IL-6, IL-8 and TNF-α significantly decreased after moderate-intensity exercise (compared with the SG, *p* < 0.05; Fig. 1I–L).

### Exercise downregulated Wnt/β-catenin and upregulated PPARγ

**Fig. 2:**
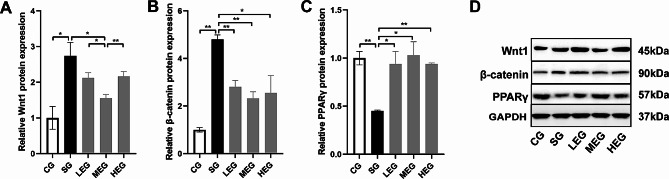
Protein expression changes after exercise training. **A**-**C**, relative protein expressions of Wnt1, β-catenin and PPARγ in lung; **D**, representative images of immunoblots of Wnt1, β-catenin and PPARγ in lung. Full-length blots are presented in Supplementary Fig. 1. CG, control group; SG, cigarette smoke group; LEG, low-intensity exercise group; MEG, moderate-intensity exercise group; HEG, high-intensity exercise group; *p < 0.05; **p < 0.01

CS significantly increased the protein expression levels of Wnt1 and β-catenin (compared with the CG, *p* < 0.05; Fig. 2A and B, and 2D), and decreased that of PPARγ in the lung (compared with the CG, *p* < 0.01; Fig. 2C and D). By contrast, exercise training at different intensities significantly reduced the protein expression levels of β-catenin and increased those of PPARγ (compared with the SG, *p* < 0.05; Fig. 2B and C, and 2D). In particular, moderate-intensity exercise significantly decreased the Wnt1 protein level (compared with SG, *p* < 0.05; Fig. 2A and D).

### Exercise combined with wnt antagonist improved COPD

**Fig. 3:**
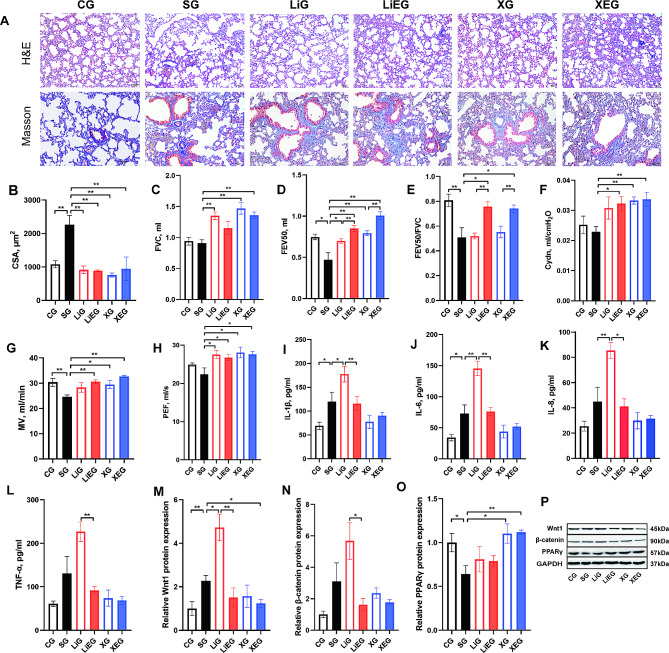
Exercise improved COPD through Wnt/β-catenin signaling. **A**, lung sections stained with HE and Masson (Bar = 50 μm, 100× and 200×, respectively); **B**, CSA of alveolar; **C**–**H**, parameters of lung volume and ventilation function; **I**–**L**, cytokine levels of BALF; **M**–**O**, relative protein expressions of Wnt1, β-catenin and PPARγ in the lung; **P**, representative images of immunoblots of Wnt1, β-catenin and PPARγ in the lung. Full-length blots are presented in Supplementary Fig. 2. CG, control group; SG, cigarette smoke group; LiG, LiCl group; LiEG, LiCl and exercise group; XG, XAV939 group; XEG, XAV939 and exercise group. *p < 0.05; **p < 0.01

To further investigate the mechanism through which exercise contributed to the improvement of COPD, we used the Wnt agonist LiCl and antagonist XAV939 combined with exercise, and the results are shown in Fig. 3. The levels of emphysema, which is indicated by alveolar CSA after drug use with or without exercise, were alleviated compared with those in the SG (*p* < 0.01, Fig. 3A and B). The results from lung function analysis show that the Wnt antagonist can reverse the decreases in FVC, FEV50, Cydn, MV, and PEF (compared with the SG, *p* < 0.01; Fig. 3C and D, Fig. 3F and H). The combination of the Wnt antagonist with exercise can restore the diminished levels of FVC, FEV50, FEV50/FVC, MV, Cydn, and PEF (compared with the SG, *p* < 0.05; Fig. 3C–H). The cytokine level results indicate that IL-1β, IL-6, and IL-8 levels were significantly aggravated after administering the Wnt agonist (compared with the SG, *p* < 0.05; Fig. 3I–K). The role of Wnt/β-catenin signaling in the improving of COPD via exercise was determined based on the abovementioned results. Given that Wnt directly regulates the PPARγ level [14], the protein expression levels of Wnt, β-catenin, and PPARγ were determined (Fig. 3M–P). The combination of the Wnt antagonist and exercise resulted in a significant increase in the protein level of PPARγ (compared with the SG, *p* < 0.05; Fig. 3O).

## Discussion

**Fig. 4:**
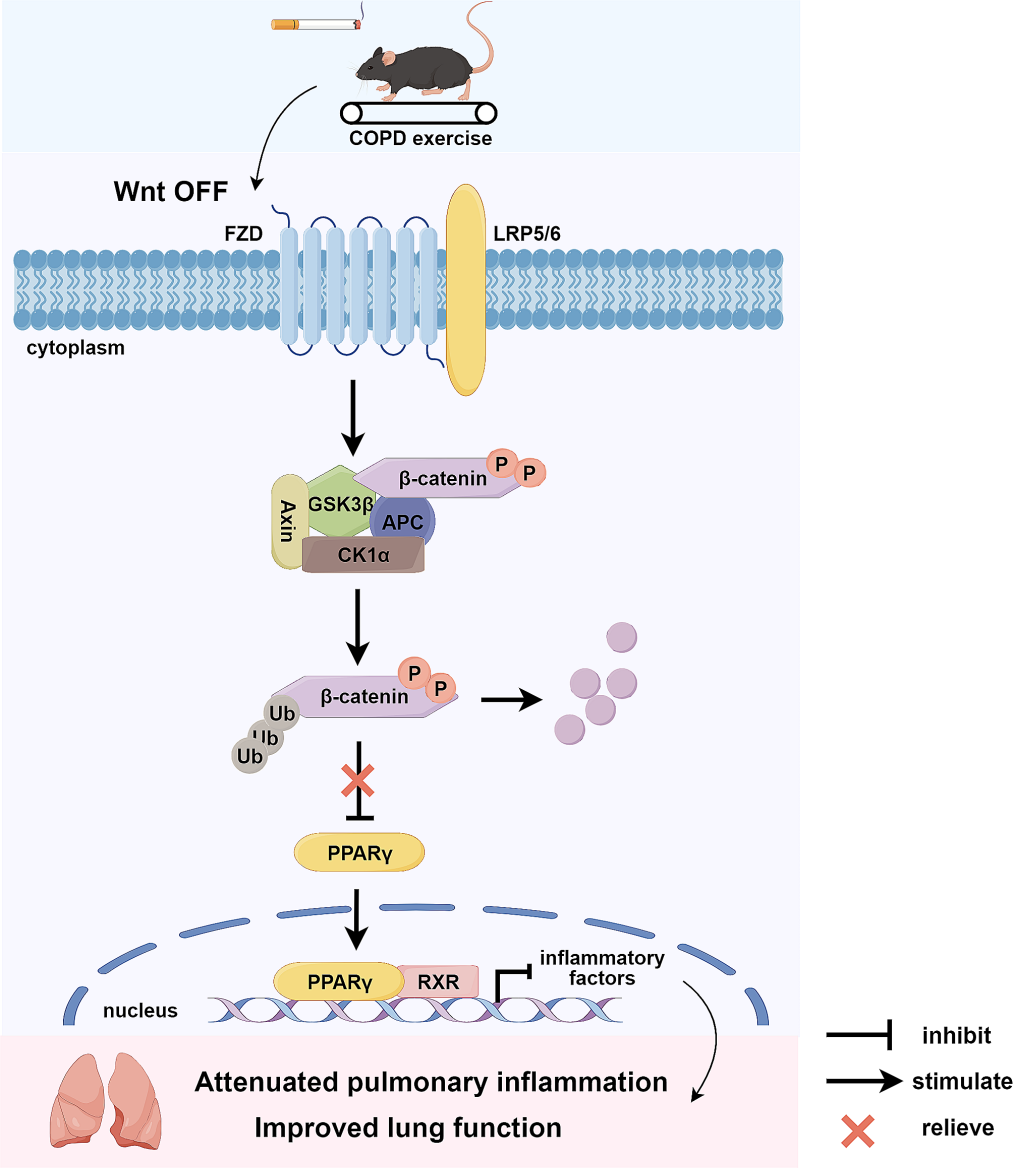
Exercise alleviated pulmonary inflammation via Wnt/β-catenin-PPARγ

In this work, we explored the effects of exercise intensity on Wnt/β-catenin pathways in a mouse model of COPD, and the results revealed the effectiveness of moderate-intensity exercise in regulating inflammatory Wnt signaling in COPD. Moreover, the combined treatment involving the Wnt antagonist and exercise more effectively alleviated pulmonary inflammation, possibly via their actions on PPARγ. These results indicate that exercise, especially moderate-intensity exercise, may exert anti-inflammatory effects partially by inhibiting Wnt/β-catenin and activating PPARγ, improving impaired lung function (Fig. 4).

Exercise can inhibit Wnt/β-catenin in COPD mice. Therefore, the degradation of β-catenin reduced its suppression of PPARγ. PPARγ can repress inflammatory pathways, such as nuclear factor κB and inflammatory factor release [33]. Subsequently, pulmonary inflammation was attenuated and impaired lung function was improved. It was generated using Figdraw (figdraw.com).

Inflammation refers to the fundamental pathophysiological changes in the lungs affected by COPD [2]. CS activates airway epithelial cells, which secrete adhesion molecules, recruit neutrophils and release elastase, and these cause damage to lung tissues [34]. Impaired airway epithelial cells can release damage-associated molecular patterns into the extracellular matrix, which target pattern recognition receptors and activate transcription factors such as nuclear factor-κB (NF-κB), to promote the release of various inflammatory substances including TNF-α, IL-6, and IL-1β, and eventually cause airway remodeling, mucus secretion, and emphysema-like injury [6, 35]. Our study revealed that, CS exposure can substantially induce lung inflammation, emphysema, and damage pulmonary function in mice. Regular exercise at appropriate intensity exerts anti-inflammatory effects, with 12 weeks of moderate-intensity aerobic exercise significantly reducing the plasma levels of IL-2, IL-4, IL-6, TNF-α, and C-reactive protein (CRP) in COPD patients [19]. In a prior study, patients with severe COPD exercised at 85% maximum exercise speed for 7 weeks. Their maximum exercise capacity was significantly increased, but the levels of serum inflammatory factors IL-6, CRP, and TNF-α showed no significant change [36]. These findings are consistent with our results. We used moderate-intensity exercise combined with a Wnt agonist and antagonist to illuminate further the mechanism of exercise in alleviating inflammation in COPD mice. We utilized moderate-intensity exercise for the following reasons: First, moderate-intensity exercise positively affects almost all pulmonary function parameters except for PEF and exhibits greater effectiveness in the alleviation of MV compared with low- and high-intensity exercises. Second, low- and moderate- intensity exercises can alleviate BALF inflammation. Moderate-intensity exercise decreased the IL-6 and TNF-α levels more significantly compared with low-intensity exercise. Moreover, high-intensity exercise failed to alleviate inflammation and exacerbated inflammation compared with moderate-intensity exercise. Finally, moderate-intensity exercise significantly affected Wnt expression compared with low- and high-intensity exercises. For these reasons, a moderate-intensity exercise was selected for the training protocol.

We determined the role of Wnt/β-catenin in mediating the improvement in the pulmonary inflammation and pathological hallmarks in a mouse model of COPD through exercise. Extracellular Wnt proteins bind to corresponding receptors on the cell membrane, which prevents the phosphorylation and ubiquitination of β-catenin. As a result, β-catenin can accumulate in the cytoplasm and translocate to the nucleus to bind to TCFs/LEFs. Thus, the downstream target genes of the pathway can be successfully transcribed [37, 38]. Consistent with our findings, Carlier et al. observed that the Wnt antagonist increased ciliated cell numbers, epithelial polarity, and barrier function to revert COPD. β-catenin shows an inverse correlation with pulmonary function in COPD patients [39]. However, studies have also proposed decreased β-catenin levels in the lungs of patients affected by COPD [40]. CS extract decreased Wnt levels in BEAS-2B cells [41], and the downregulated Wnt targeted genes in airway epithelial cells [42]. Intraperitoneal injection of LiCl into COPD mice led to decreased mean linear intercept levels and improved dynamic compliance of the lung [43]. In three-dimensional (3D) COPD lung tissues, the Wnt agonist decreased the level of MMP12 and alleviated elastin deposition in the alveolar wall [44]. The difference possibly resulted from model differences (CS exposure and intratracheal elastase installation, in vitro and in vivo), sampling (whole lung and airway), and the dual role of Wnt in inflammation (Wnt stimulates inflammation [45] and inhibits inflammation [46]). Similarly, opposite changes in Wnt occurred in various organs after exercise. Exercise activated Wnt/β-catenin to promote skeletal muscle regeneration, improve neurogenesis and myelin repair, and delay the early healing of rotator cuff injury [24, 47, 48]. Exercise also inhibited Wnt/β-catenin in neurons of sciatic nerve injury rats and in the plasma of breast cancer patients [49, 50]. In one study, maternal swimming improved the memory function of pups by inhibiting Wnt/β-catenin [51]. We observed that low-, moderate- and high-intensity aerobic exercise can decrease β-catenin levels. Moderate-intensity exercise also decreased the Wnt protein levels. The above studies indicate that various factors may influence the regulatory effect of Wnt on COPD, but its role remains inconclusive. In our study, the Wnt antagonist promoted exercise ameliorated COPD pulmonary inflammation, and improved lung function.

Wnt/β-catenin inhibits PPARγ activity through a direct interaction between TCF/LEF binding domain of β-catenin and catenin binding domain of PPARγ [13]. The combination of β-catenin and PPARγ lowers the activation of the PPARγ ligand on PPARγ [52]. The target gene of TCF/LEF, cyclin D1, can inhibit the activity of PPARγ [53]. In addition, PPARγ interacts with β-catenin through pathways, such as those of the signal transducer and activator of transcription 3 and epidermal growth factor receptor - phosphoinositide-3-kinase-Akt [54]. Previous studies have demonstrated decreased PPARγ levels in COPD patients and animals [15, 55]. CS-extract-treated bronchial epithelial cells showed time- and dose-dependent decreases in PPARγ protein levels [56]. PPARγ of COPD shows a positive correlation with FEV_1_%, and a negative association with the residual volume and the ratio of residual and lung volumes [15]. Moreover, PPARγ ligands can inhibit CS-induced inflammation of the lungs, airway smooth muscle cells, and epithelium [17, 57, 58], and alleviate emphysema [59]. Airway epithelium PPARγ conditional deletion mice exhibited a higher macrophage count than wild-type mice after smoke exposure [60]. Consistent with this previous finding, our study demonstrated that 25 weeks of CS exposure significantly decreased the PPARγ protein level. In addition, exercise of different intensities can upregulate PPARγ. Regular exercise can also upregulate PPARγ and its receptor, which decreases the levels of IL-1β [61, 62]. In our study, the combination of exercise with Wnt antagonists also significantly increased the level of PPARγ in the lung tissue, but the Wnt agonist showed no effect. Other downstream signaling targets of Wnt signaling, such as NF-κB and cAMP responsive element-binding protein (CREB), may mediate the effect of Wnt on COPD. NF-κB is a crucial inflammatory factor negatively or positively regulated by Wnt signaling [63]. Moreover, studies have shown that exercise regulates NF-κB [64, 65]. In addition, CREB is a coactivator of Wnt/β-catenin-mediated transcription, and LiCl promotes CREB activation [66, 67]. CREB can competitively bind with and inhibit NF-κB, which results in downregulated inflammation [68]. Studies also revealed that exercise upregulated the total CREB and phosphorylation levels at ser133 [69, 70]. Previous research indicated the upregulation of the non-canonical Wnt signaling pathway in COPD. In mice and human 3D models of COPD, CS upregulated the non-canonical Wnt5a and inflammation [71]. Lung-specific Wnt5a overexpression exacerbated alveolar enlargement in COPD mouse models [72]. The recombinant Wnt5a and Wnt5b can promote the release of IL-6 and IL-8 release from human lung fibroblasts [73]. However, the effect of exercise on the non-canonical Wnt pathway has not been determined. Thus, whether the effect of exercise on COPD is correlated with the non-canonical Wnt pathway remains unclear. Further studies are needed to elucidate the exact mechanism underlying exercise-alleviated COPD.

## Strengths and limitations

As described in the Discussion, CS is a significant risk factor for COPD. CS, as the most commonly-used COPD model construction medium, can induce chronic bronchitis and emphysema [49]. Therefore, one strength of our study is the classic and solid COPD model. Pulmonary function test is one of the diagnosis standards and severity assessments used for diagnosis and management of COPD. Another strength of our study is the use of Buxco pulmonary function test systems to gain lung volume and ventilation. The limitations of our study include its small sample size and time-consuming CS protocol.

## Conclusion

Aerobic exercise, especially moderate-intensity exercise, can alleviate CS-induced pulmonary function impairment, COPD-like pathophysiological changes and pulmonary inflammation infiltration. The effects of exercise may be related to the inhibition of Wnt/β-catenin and therefore the activation of PPARγ in the lungs of COPD.

### Electronic supplementary material

Below is the link to the electronic supplementary material.

Supplementary Material 1: Fig. 1. Full-length blots of Fig. 2D

Supplementary Material 2: Fig. 2. Full-length blots of Fig. 3P

## Data Availability

The datasets used and/or analyzed during the present study are available from the corresponding author on reasonable request.

## Abbreviations

BALF: Bronchoalveolar lavage fluid
COPD: Chronic obstructive pulmonary disease
CS: Cigarette smoke
Cydn: Dynamic lung compliance
FEV: Forced expiratory volume
FVC: Forced vital capacity
IL: Interleukin
MV: Minute ventilation
PPARγ: Peroxisome proliferator-activated receptor γ
PEF: Peak expiratory flow
TNF-α: Tumor necrosis factor-α
