# Supplementary figures and images for: Aerobic exercise training engages the canonical wnt pathway to improve pulmonary function and inflammation in COPD
Source: BMC Pulm Med. 2024 May 14;24:236. doi: 10.1186/s12890-024-03048-z (PMC11095004; doi:10.1186/s12890-024-03048-z)

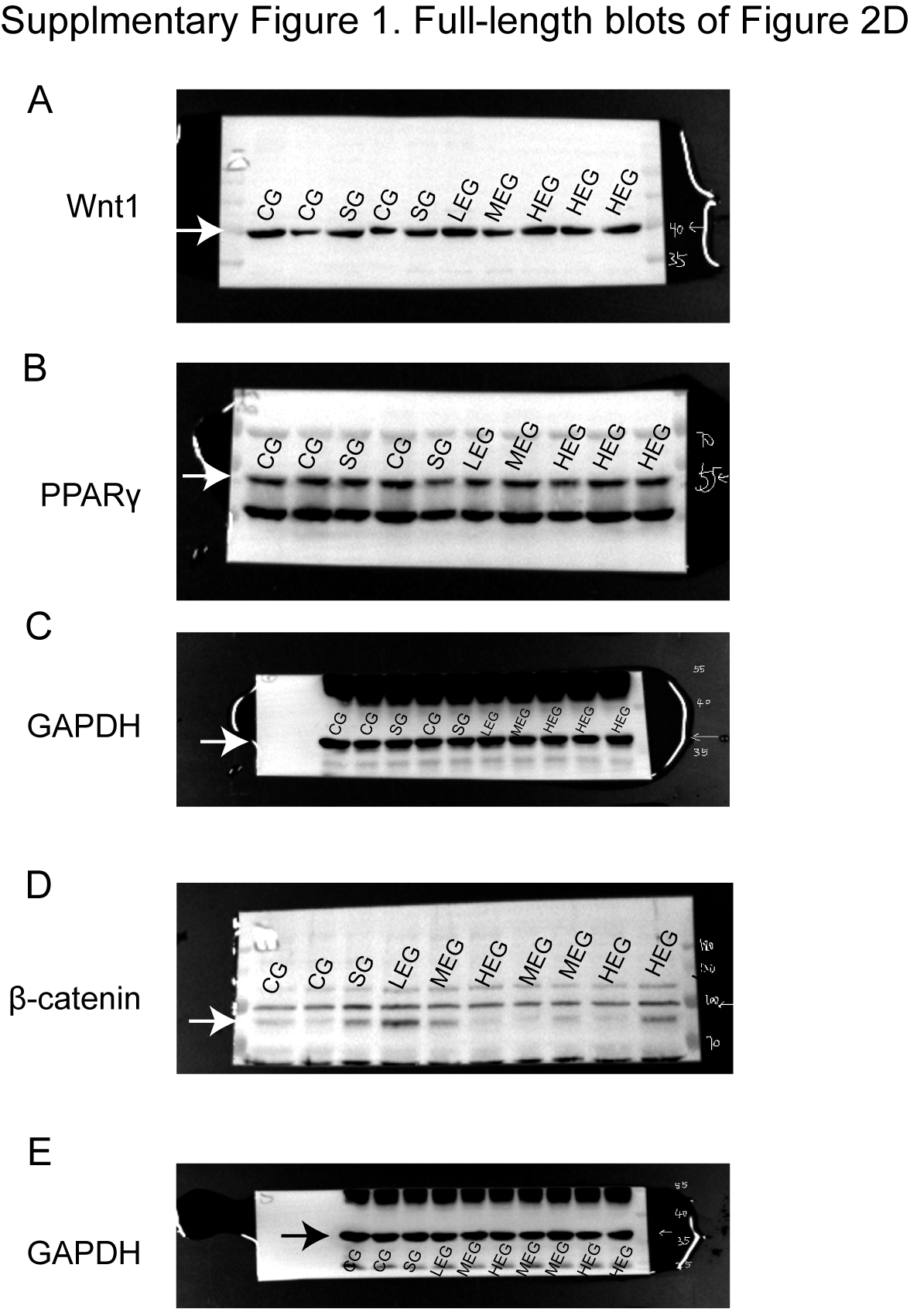

Supplement: Supplementary file 1 — Supplementary Material 1: Fig. 1. Full-length blots of Fig. 2D [file 12890_2024_3048_MOESM1_ESM.tif]

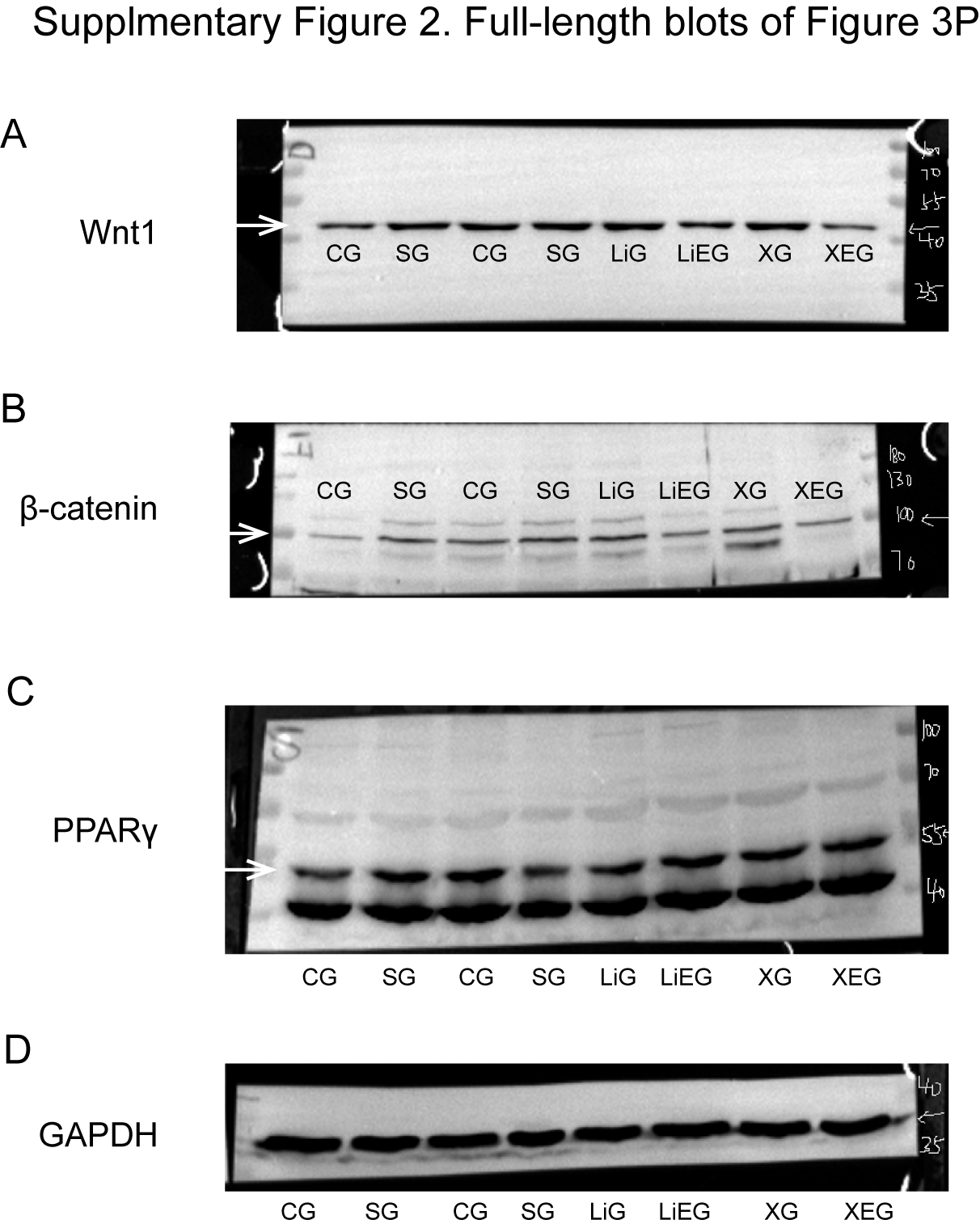

Supplement: Supplementary file 2 — Supplementary Material 2: Fig. 2. Full-length blots of Fig. 3P [file 12890_2024_3048_MOESM2_ESM.tif]
